# A comparison of maceration methods for the preparation of infant skeletal remains for forensic anthropological analysis

**DOI:** 10.1007/s00414-023-03137-4

**Published:** 2023-11-22

**Authors:** C. A. Keyes, K. R. Giltrow, T.-J. Mahon

**Affiliations:** Department of Forensic Medicine and Pathology, School of Clinical Medicine, Faculty of Health Sciences, University of the Witwatersrand, Johannesburg, South Africa

**Keywords:** Maceration, Forensic anthropology, Juvenile, Skeleton, Bones, Detergent

## Abstract

Very little literature currently exists prescribing which maceration method to use when preparing infant human remains, resulting in bone quality that is suitable for forensic anthropological analysis. The aim of the study was to test five maceration methods to determine which is most suitable for infant remains for forensic anthropological analysis. The sample included five neonate pig carcasses (*Sus scrofa domesticus)*, ranging between one to three days old. Five maceration methods were tested on the pig carcasses (one pig per maceration method) to determine their effectiveness. The methods included invertebrate maceration by meal worms, chemical maceration by bleach, chemical maceration by borax solution, enzymatic maceration by laundry detergent and sodium carbonate solution, and chemical maceration by sodium hypochlorite. A scoring method was created to assess the effectiveness of each maceration method. Invertebrate maceration and chemical maceration using bleach were the least successful methods of maceration (total maceration score = 8 respectively). Chemical maceration using borax and chemical maceration using sodium hypochlorite achieved complete maceration of the skeletal remains; however, they both resulted in artifacts that are unsuitable for forensic analysis (total maceration score = 14 respectively). Enzymatic maceration using laundry detergent and sodium carbonate was the most successful method (total maceration score = 17). The detergent technique subsequently successfully macerated all five sets of infant human remains. This study has validated that the enzymatic maceration technique using laundry detergent and sodium carbonate can be used to effectively macerate the remains of infant skeletal remains for forensic anthropological analysis.

## Introduction

Maceration, which is the process of cleaning bones by removing soft tissues from bone surfaces, is used in numerous scientific fields. This allows for the analysis and preservation of bones in forensic investigations, for display in museum collections, or taxidermy [1]. Several maceration methods are commonly used, which can be grouped into six categories: warm water bacterial maceration, cold water bacterial maceration, hot water maceration, chemical maceration, enzymatic maceration, and invertebrate maceration [1–6].

Each maceration method has its own advantages and disadvantages, which must be weighed and selected according to its intended purpose and the properties of each skeletal case. Practitioners must consider several factors when selecting the appropriate maceration method for their intended use of bones or the present state of the bones. For example, frozen bones, burned bones, bones with trauma, and infant, sub-adult, and adult bones all have different mechanical properties, and the selected maceration method must be suitable for the unique context of each case [7]. Practitioners must take care when macerating remains because the maceration method may have abrasive qualities with the potential to create postmortem modifications or alter vital existing evidence such as trauma or create modifications that can be misinterpreted as peri-mortem trauma [8]. Additional factors that must also be considered include the ease of the method, the time taken to complete the process [1], the resources required or available for the process, the potential safety risks of chemicals used [1], the resulting bone quality and color obtained, as well as the odor produced [1, 6]. Practitioners will weigh these factors differently depending on their required outcome and available resources.

Forensic anthropologists are frequently required to analyze infant human remains, especially for the estimation of age-at-death, which can be determined reliably through close examination of the dentition, morphology, and ossification centers of the skeleton [9]. Infant bones have unique physiochemical properties—notably the greater proportion of immature or woven bone with a larger composition of organic material compared to adult bones—which result in them being relatively fragile and difficult to preserve [9]. As a result, the maceration of infant remains is challenging and must be performed with caution such that the ability to identify and assess even the smallest of the infant bones is not hampered in any way.

Most published literature on various maceration methods is applied to adult skeletal remains, but very little literature currently exists prescribing which maceration method to use when preparing infant human remains [10]. Determining which technique is most suitable for the removal of soft tissues from infant human remains will ensure that future macerated specimens are in a condition good enough to undergo a thorough forensic anthropological analysis. The aim of the present study was to test several maceration methods to determine which is most suitable for infant remains for forensic anthropological analysis.

## Methods

This study was conducted at the Forensic Anthropology Laboratory of the Johannesburg Forensic Pathology Service Medico-legal Mortuary. Ethical clearance was provided by the Animal Ethics Screening Committee (clearance number 2013/19/01) and Human Research Ethics Committee (clearance number M130414).

**Fig. 1:**
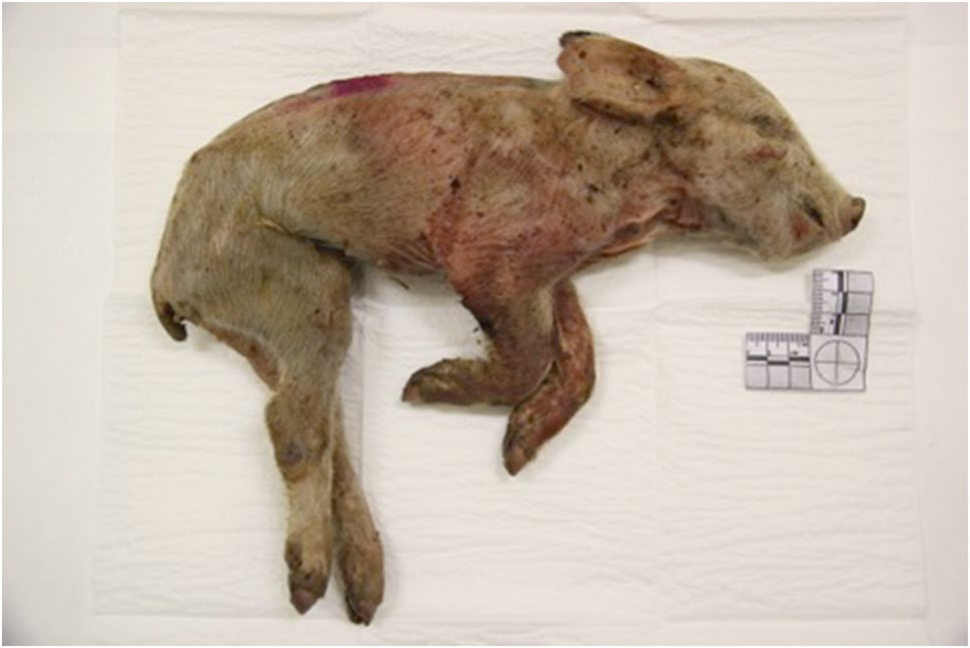
A neonate porcine carcass used to test maceration methods

The sample included five neonate pigs (*Sus scrofa domesticus)*, ranging between one to three days old (Fig. 1). The pigs died from natural causes and were donated to the study by a local pig farm. No animals died for the purpose of this study. Pig carcasses were used as human proxies to test five maceration methods before isolating the best method, which was later tested on human infant remains. Pigs are a common animal model used as human analogues in forensic anthropological studies because porcine bone displays similarities in anatomy, morphology, healing, remodeling, density, and mineral concentration with human bone [11].

The sample also included the fleshed remains of five deceased full term human neonates that underwent routine medico-legal autopsies at the Johannesburg Forensic Pathology Medico-Legal Mortuary. These remains were unclaimed and unidentified neonates that remained unclaimed after thirty days. Decedents that remain unclaimed after 30 days are typically buried by the municipality; however, these remains were approved for use in the study due to its benefit for future cases that will require maceration and forensic anthropological analysis. The skeletal remains were then archived in a forensic anthropology repository for unidentified unclaimed remains up until the time that they are identified and claimed.

The neonate pig carcasses were placed in a mortuary fridge for 30 days prior to maceration, to recreate the state of decomposition as the unclaimed infant human remains. All remains were skinned and eviscerated prior to their undergoing maceration.

### Maceration methods

Five maceration methods were tested on the pig carcasses (one pig per maceration method) to determine their effectiveness. The methods included invertebrate maceration by meal worms, chemical maceration by bleach, chemical maceration by borax solution, enzymatic maceration by laundry detergent and sodium carbonate solution, and chemical maceration by sodium hypochlorite. The length of time for each method varied and depended on how often the macerating agent required replacement. Due to the fragile nature of the juvenile bones, an additional defatting process was not implemented after the maceration process as is usually done with adult bones. A summary of the maceration processes are tabulated in Table 1.

**Table 1:** Comparison of maceration methods setup

Method	Products	Setup	Maceration method duration	Post-maceration manual bone cleaning time	Post-maceration drying
Invertebrate maceration	Live meal worms, plastic container, bran bedding medium	Remains were disarticulated and placed in the plastic container. Remains were covered with bran bedding medium. Meal worms were placed in the container, covering the remains	3 weeks	Not applicable	Not applicable
Chemical maceration using bleach	Generic household bleach, stainless-steel pot	Remains were disarticulated and placed in the stainless-steel pot. Bleach was added until the remains were submersed. Bleach replaced every 15 min	60 min	Not applicable	Not applicable
Chemical maceration using sodium hypochlorite	Sodium hypochlorite (SANS 10228), stainless-steel pot	Remains were disarticulated and placed in the stainless-steel pot. Sodium hypochlorite was added until the remains were submersed	5 min	Not required	Bones were rinsed with tap water and allowed to dry thoroughly at ambient temperature beneath a fan
Chemical maceration using borax solution	Stainless-steel pot, gas stove, borax solution with a concentration of 100 mL of powdered borax per 1L of water	Remains were disarticulated and placed in the stainless-steel pot. Borax solution (heated to 75 °C) at was added until the remains were submersed. Borax solution retained at simmering temperature over a gas stove	90 min	85 min	Bones were rinsed with tap water and allowed to dry thoroughly at ambient temperature beneath a fan
Enzymatic maceration using laundry detergent and sodium carbonate solution	Stainless-steel pot, gas stove, enzyme solution with a concentration of 20 mL of powdered laundry detergent with active enzymes and 20 mL of sodium carbonate powder per 2 L of water	Remains were disarticulated and placed in the stainless-steel pot. Enzyme solution (heated to 75 °C) at was added until the remains were submersed. Enzyme solution retained at simmering temperature over a gas stove	4 h	135 min	Bones were rinsed with tap water and allowed to dry thoroughly at ambient temperature beneath a fan

#### 1] *Invertebrate maceration*

Invertebrate maceration using meal worms [12] was achieved by placing the carcass into a plastic container of meal worms, which were covered with a bran bedding medium. The consumption of the carcass by the meal worms was reviewed every 24 h.

#### 2] *Chemical maceration*

Chemical maceration using bleach [4] was achieved by disarticulating the pig carcass and immersing the remains in undiluted household bleach in a stainless-steel pan for 15-min intervals. The bubbling of the bleach and resulting heating up of the liquid indicated that the soft tissues were being chemically removed. After the completion of each interval, the remains were lifted from the bleach to monitor the progress of the maceration. Before being placed back into the pan for the next 15-min interval. During each interval, the used bleach was discarded and replaced with fresh household bleach.

Chemical maceration using sodium hypochlorite (SANS 10228) [13] was achieved by placing the disarticulated pig carcass in a stainless-steel pot, and the remains were immersed in sodium hypochlorite. Progress of the chemical maceration was checked at 15-min intervals.

Chemical maceration using a borax solution [14] was achieved by placing the disarticulated pig carcass into a stainless-steel pot and immersed in a solution with a concentration of 100 mL of powdered borax per 1 L of water. This chemical maceration method was combined with warm water maceration; therefore, the temperature of the solution was preheated and then maintained at a temperature of 75 °C and allowed to simmer, with progress of the maceration checked at 15-min intervals.

#### 3] *Enzymatic maceration*

Enzymatic maceration using laundry detergent and sodium carbonate solution [1, 15] was achieved by placing the dismembered pig carcass into a stainless-steel pot and immersed in a solution with a concentration of 20 mL of powdered laundry detergent with active enzymes and 20 mL of sodium carbonate powder per 2 L of water. This enzymatic maceration method was combined with warm water maceration to aid the enzyme activity; therefore, the temperature of the solution was pre-heated and then maintained at a temperature of 75 °C and allowed to simmer, with progress of the maceration checked at 15-min intervals.

After each interval, the remains were removed from their maceration medium to determine if the soft tissues were soft enough for manual removal of the soft tissues from the bones by brushing. If bones could not be removed from the soft tissues, they were returned into their maceration medium for a new interval. This was repeated until maceration had been completed. Thereafter, the porcine bones were rinsed with tap water and allowed to dry thoroughly at ambient temperature beneath a fan.

### Assessment of maceration methods

A scoring method was created to assess the effectiveness of each maceration method. As outlined in Table 2, numerical scores were assigned to common observations seen in all the tested maceration methods. A score ranging from 1 to 5 was assigned to four variables assessed in each method: soft tissue breakdown, bone exposure, bone condition, and odor intensity. The scores were assigned after maceration was completed. The four scores assigned to each sample were added for a total score, which indicates the overall effectiveness of the maceration method. Higher scores indicate greater effectiveness of the respective method. The maceration method that scored the highest was then repeated on five human infant remains to validate its effectiveness in human remains.

**Table 2:** Scoring system developed for the description of soft tissue breakdown, bone exposure, bone damage, and odor intensity produced during maceration

Score	Soft tissue breakdown descriptions	Bone exposure descriptions	Bone condition descriptions	Odor intensity descriptions
5	No soft tissues attached	All bones are exposed All bones are disarticulated	No damage is observed	Faint odor, not unpleasant and barely perceptible
4	Soft tissues have become loose and jelly-like in consistency. Cartilage is soft. Tissues can be scraped from the bones	Upper limb bones, scapulae, pelvis, and tips of the vertebral processes becoming exposed. Bones can be pulled out of the soft tissues	Bones beginning to appear chalky	Faint odor, slightly unpleasant but not interfering with comfort
3	Soft tissues are still attached to bone. Tissues are beginning to soften. Some tissues can be pulled off bones	Bones becoming exposed in areas where soft tissue layer covering them was relatively thin. Parietal bones, mandible, ribs, and lower parts of the limbs becoming exposed	Bones appear very chalky. Bones are beginning to soften	Noticeable odor that is distracting and interferes with comfort
2	Soft tissue is still attached to bone. Tissues are becoming a “cooked” color. Tissues are beginning to shrink	Ends of the ribs becoming exposed	Bones appear extremely chalky. Ends of the long bones beginning to flake. Bones becoming eroded	Strong odor that is very distracting and causes discomfort
1	All soft tissues are still attached to bone. No breakdown of the soft tissues is observed	No bones are exposed	Bones are fragile and soft OR Bones are not observable due to soft tissue adherence	Very unpleasant odor that is strong enough to cause a physiological response

## Results

Invertebrate maceration (meal worms) and chemical maceration using bleach were the least successful methods of maceration (total maceration score = 8 for both methods) (Table 3). The invertebrates did not consume any soft tissues, resulting in no breakdown of the tissues and no bone exposure. Invertebrate maceration was concluded after three weeks once the meal worms had died without consuming any soft tissues.

**Table 3:** Comparison of scores indicating the effectiveness of five maceration methods

	Invertebrate (meal worms)	Chemical (bleach)	Chemical (sodium hypochlorite)	Chemical (borax)	Enzymatic (laundry detergent and sodium carbonate)
Soft tissue breakdown score	1	2	5	4	4
Bone exposure score	1	3	5	4	4
Bone condition score	1	2	3	2	5
Odor intensity score	5	1	1	4	4
Total score	8	8	14	14	17

Chemical maceration resulted in minimal softening and removal of some soft tissues, exposing tendons and minimal exposure of the tibiae, fibulae, ribs, vertebral bodies, mandible, and parietal bones. The bleach resulted in the exposed bones developing a chalky appearance and erosion of the exposed rib surfaces. The fumes produced by the bleach (despite ventilation) resulted in an increasing unpleasant odor intensity that resulted in a physiological response in the form of eye irritation. Due to the hazardous nature of the bleach maceration, with no significant perceived change in the soft tissues, this maceration method was concluded after 60 min.

Chemical maceration using borax and chemical maceration using sodium hypochlorite achieved complete maceration of the skeletal remains; however, they both resulted in artifacts that are unsuitable for forensic analysis (total maceration score = 14 for both methods) (Table 3). Borax maceration was completed in 3 h (90 min simmering time; 85 min manual cleaning time) and resulted in the soft tissues developing a gelatinous consistency that could easily be removed from the bones. Although cartilage remained attached to the bones, it could be easily removed with light brushing and manual removal. The vertebral column remained articulated and required manual disarticulation. With ventilation, the odor produced by borax maceration was very minimal and only perceptible close to the sample. After maceration and drying, all bones were identifiable, disarticulated, and not greasy, with no apparent odor. The bones were a creamy color and complete enough for accurate measurements to be taken. Minimal soft tissues remained attached to the bones. However, borax maceration was not a suitable method due to the significant damage it caused to the bones, in the form of tooth cracking, cortical bone flaking and erosion, and exposure of spongy bone. Borax maceration was also the costliest method of maceration.

Sodium hypochlorite maceration was the most rapid method, resulting in complete maceration and disarticulation in 15 min, with non-greasy bones that require no manual cleaning of soft tissues. However, sodium hypochlorite creates dangerous fumes requiring the technician to use a gas mask, which does not entirely remove the perception of the strong chlorine smell. The resulting bone quality was poor, with bones developing a chalky appearance, and the structure was compromised resulting in the bones becoming soft and fragile. Sodium hypochlorite maceration was the second most costly method of maceration.

**Fig. 2:**
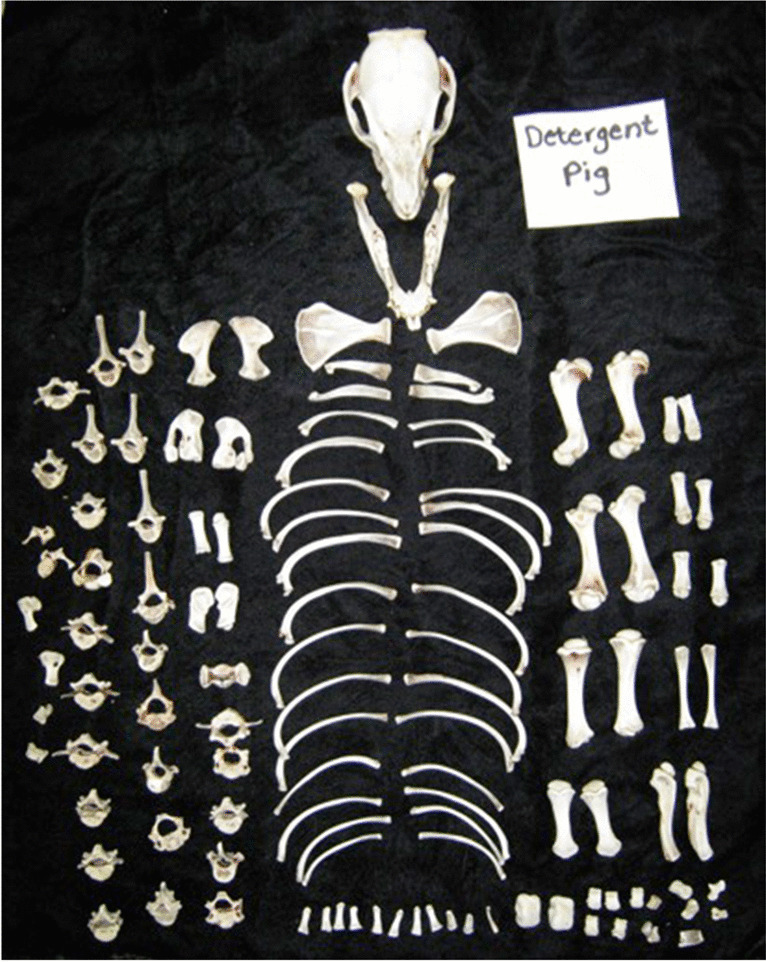
Neonate pig skeleton successfully macerated using the laundry detergent method

Enzymatic maceration using laundry detergent and sodium carbonate was the most successful method (total maceration score = 17) (Table 3); however, it was the longest method of maceration (4 h: 105 min simmering time; 135 min manual cleaning time). This method resulted in the soft tissues developing a gelatinous consistency that allowed bones to disarticulate and be easily removed. Minimal soft tissue still adhered to the bones, but they could easily be removed by light brushing and rinsing in water. This method resulted in a mild, relatively-unoffensive odor. No damage was observed on the bones after maceration and drying, where white in color, and retained the odor of the laundry detergent (Fig. 2). The consumables for this maceration method were considerably cheaper than the other methods.

**Fig. 3:**
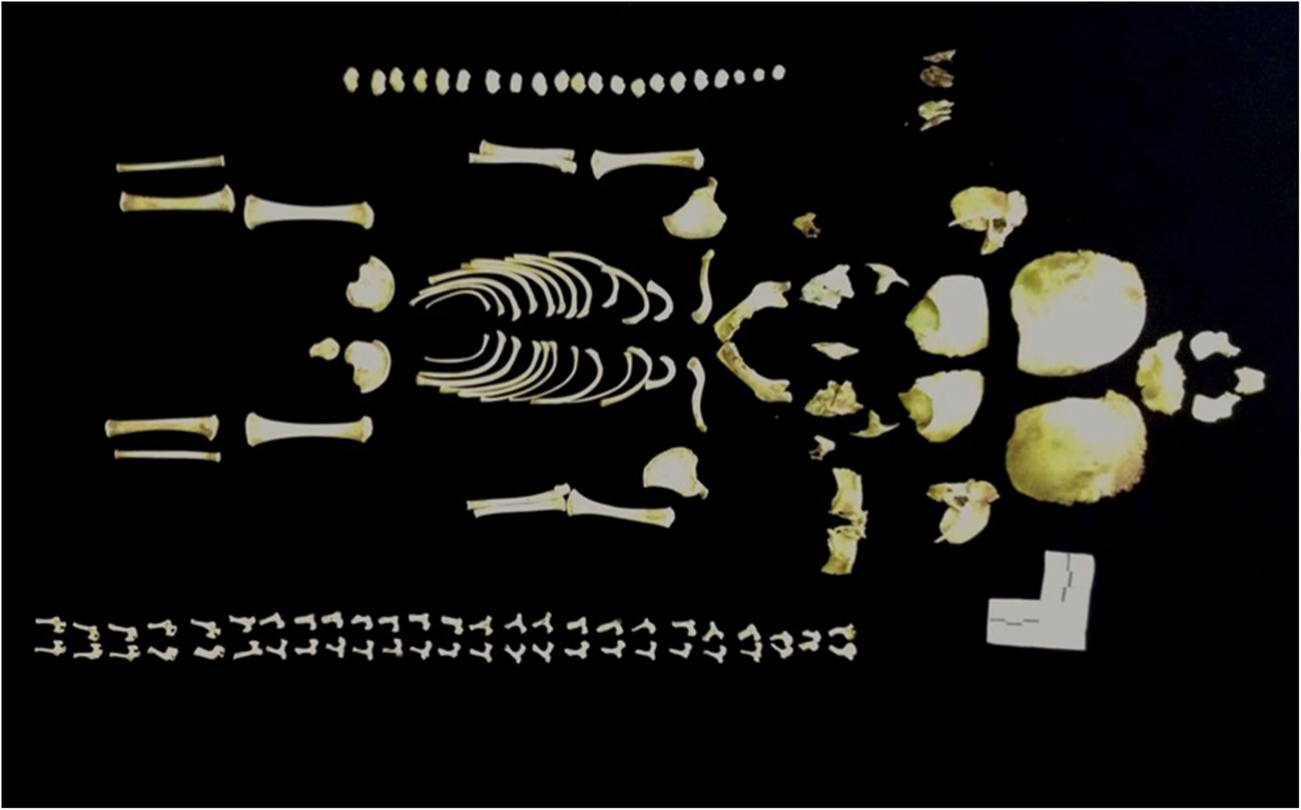
Infant human skeleton successfully macerated using the laundry detergent method

Due to the success of the enzymatic maceration method using laundry detergent and sodium carbonate on neonate pig carcasses, this method was further tested on five infant human remains to validate its use for forensic cases. The detergent technique successfully macerated all five sets of infant human remains. Complete disarticulation of all skeletal elements was achieved in all specimens except one case (which only had two pairs of centra remaining articulated). All the resulting skeletal specimens were non-greasy, disarticulated, identifiable, and complete, allowing for accurate measurements to be taken, with no unpleasant bone odor (Fig. 3).

Minimal soft tissues were present on all specimens following the completion of maceration, which were not removable. One case had small quantities of soft tissue only present on the processes of the vertebral arches, one case had soft tissues noted on the ischium, and one case had soft tissues covering both sides of the ethmoid bone. Four of the cases had soft tissues present on cranial and vertebral bones. In terms of cartilage, only one case was completely free of adhered cartilage. The other four cases had cartilage present on the vertebral centra.

There was no damage noted to the skeletal elements in one case, but minor damage to the osseous surfaces was recorded on four cases, including erosion of the pars petrosa (in one case) and vertebral centra (in two cases), and discoloration of the epiphyseal ends of the long bones (in four cases). One case exhibited fragmentation of the right ethmoid bone into two fragments.

## Discussion

Several maceration methods that are commonly used for adult skeletal remains or suggested in previous literature were tested for their efficacy on infant remains. Most methods proved to be too harsh or ineffective in producing skeletal specimens of suitable quality for forensic anthropological analysis. The enzymatic maceration technique using laundry detergent and sodium carbonate [15] was the most successful maceration method overall in both the neonate pig controls and the human infant remains, and the authors suggest that this method be used for forensic cases. Despite the general success of the detergent maceration method, there were several limitations that practitioners should be aware of.

The detergent maceration method included simmering as a means of hot water maceration to initiate the enzymatic action. The simmering process was easy and was completed in only a few hours. After 2 h of simmering, the tissues of the specimens were already soft enough for bones to be easily pulled free. This was likely a result of the proteolytic action of the enzyme-active ingredients found in the laundry detergent, which breaks down the structural chains of the proteins found in soft tissues [15]. This may have been further aided by the hot water, which increases the activity of preexisting bacteria in the tissues that also degrades proteins [1]. This method did have the longest duration of all the maceration methods tested, requiring numerous simmering periods. The authors suggest the use of muslin drawstring bags during the simmering to hold the specimens, which ensures that all skeletal elements were easily recovered, with no accidental loss of bones during drainage of the detergent and carbonate solution. Using these bags aids in the speed of future macerations, as more than one case can be simmered at a time, using the same amount of detergent. Overall, maceration by enzymatic action in detergent is the method recommended for future use in juveniles’ remains, and other studies have indicated its usefulness in other fleshed remains and burned remains [7, 16].

Although maceration softens and removes most soft tissues, the bone still requires subsequent manual cleaning of the bones to remove any adhering tissues, such as the cartilage, using toothbrushes of various bristle strengths. The manual cleaning of bones is extremely tedious [17] and exacerbated in infant bones due to their small size and their greater number of skeletal elements [9]. The use of manual force was often required to disarticulate the centra and scrape off the cartilage, and this was the likely cause of the erosion noted on the centra of two specimens. The amount and structure of infant cranial bones also present some difficulties during manual cleaning, with many of them being thin and fragile. The wrinkled surfaces of the ethmoid bones in one specimen were difficult to clean, and the bone was accidentally fractured during this process. As a result, brushing of the labyrinths was not completed, and soft tissues remained adhered to all surfaces of this bone. Practitioners must be careful not to cause damage to the bones when becoming tired during the manual cleaning process. Injury to the macerator can also easily occur. An ideal maceration method would eliminate these periods of manual cleaning entirely; however, manual cleaning was still required in all maceration methods and is inevitable.

Detergent maceration also resulted in discoloration of the long bone epiphyses, which was not visible until after drying, and practitioners must be aware of this artifact, although it does not impact the forensic analysis of the remains. Although some soft tissues did remain attached to the specimens, this did not hamper their assessment in any way and would not present difficulty when examining them in a forensic context. This tissue attachment would need to be resolved when preparing specimens for their inclusion in a skeletal collection for display purposes.

An additional benefit to the detergent maceration method is the minimal and inexpensive equipment required which was easily obtainable. Unlike the chemicals used in the other maceration methods, detergent presents no dangers in its transportation, usage, or storage. Completing the detergent maceration was safe and resulted in no malodors.

The other maceration methods proved to be inefficient or unsuitable for forensic cases. Neither meal worms nor undiluted household bleach resulted in complete maceration; therefore, their use in cleaning infant remains, especially in the forensic context, is not recommended. The sodium hypochlorite method of chemical maceration for infant remains is not suitable. While this technique was highly effective at removing the soft tissues from the skeletal elements, it resulted in the poor quality of skeletal remains which were not able to undergo anthropological analysis. The corrosive properties of the chemical (SANS 10228) damaged the porcine bones extensively and eroded the stainless-steel interior of the maceration pot and produced toxic fumes that are irritating to the eyes and nose and can damage living tissue. Therefore, sodium hypochlorite can be considered a safety hazard in a laboratory environment and should be avoided as a maceration method. The chemical maceration method using borax solution resulted in similar scores to the detergent method for soft tissue removal, bone exposure and odor; unfortunately, it resulted in greater bone damage, making the method unsuitable to analysis or preservation of skeletal remains.

A limitation of the study is that only fully fleshed remains in the fresh or early stages of decomposition were used in the sample. As a result, the authors did not compare the effects of different stages of decomposition for each of the maceration methods. This is worth investigating in future research. However, from the personal experience of the authors, the stage of decomposition does not have a significant impact on the result of the maceration process. Subsequent to this study, the authors’ standard practice has been updated to manually deflesh the remains as much as possible prior to maceration. As a result, the level of soft tissue decomposition becomes negligible. The enzymatic detergent method has proven to be the best method for macerating porcine bones in different states (including burned bone) [7, 16], and it is now the standard practice of the authors to use the enzymatic detergent method for adult human remains too. The authors note that, anecdotally, this method is best for adult human remains; however, it will be worthwhile for the present study to be replicated in the future using adult human remains.

## Conclusion

This study has validated that the enzymatic maceration technique using laundry detergent and sodium carbonate, amended from that suggested by Fenton et al. [15], can be used to effectively macerate the remains of infant skeletal remains. This method does have some limitations that need addressing. It is suggested that future studies develop adaptations to the method that could more effectively remove the cartilage and soft tissues that persist on the skeletal elements, especially on the vertebral column. If the simmering process can be adjusted to reduce the amount of manual cleaning required, less damage should occur to the bones which will result in a much higher quality skeletal specimen.

## Data Availability

Data sharing is not applicable to this article as no datasets were generated or analyzed during the current study.

## Declarations

### Disclaimers

The authors conceived this study and performed the data collection, analysis and interpretation, and the writing of this article. The manuscript, data, figures, and tables have not been previously published and are not under consideration elsewhere.
